# Ball Milling Innovations Advance Mg-Based Hydrogen Storage Materials Towards Practical Applications

**DOI:** 10.3390/ma17112510

**Published:** 2024-05-23

**Authors:** Yaohui Xu, Yuting Li, Quanhui Hou, Yechen Hao, Zhao Ding

**Affiliations:** 1 Laboratory for Functional Materials, School of New Energy Materials and Chemistry, Leshan Normal University, Leshan 614000, China; 2 Leshan West Silicon Materials Photovoltaic New Energy Industry Technology Research Institute, Leshan 614000, China; 3 College of Materials Science and Engineering, National Engineering Research Center for Magnesium Alloys, National Innovation Center for Industry-Education Integration of Energy Storage Technology, Chongqing University, Chongqing 400044, China; 4 School of Automotive Engineering, Yancheng Institute of Technology, Yancheng 224051, China; 5 Department of Computer Science, Illinois Institute of Technology, Chicago, IL 60616, USA

**Keywords:** magnesium-based hydrides, ball milling, nanostructures, hydrogen storage, catalysts, nanocomposites

## Abstract

Mg-based materials have been widely studied as potential hydrogen storage media due to their high theoretical hydrogen capacity, low cost, and abundant reserves. However, the sluggish hydrogen absorption/desorption kinetics and high thermodynamic stability of Mg-based hydrides have hindered their practical application. Ball milling has emerged as a versatile and effective technique to synthesize and modify nanostructured Mg-based hydrides with enhanced hydrogen storage properties. This review provides a comprehensive summary of the state-of-the-art progress in the ball milling of Mg-based hydrogen storage materials. The synthesis mechanisms, microstructural evolution, and hydrogen storage properties of nanocrystalline and amorphous Mg-based hydrides prepared via ball milling are systematically reviewed. The effects of various catalytic additives, including transition metals, metal oxides, carbon materials, and metal halides, on the kinetics and thermodynamics of Mg-based hydrides are discussed in detail. Furthermore, the strategies for synthesizing nanocomposite Mg-based hydrides via ball milling with other hydrides, MOFs, and carbon scaffolds are highlighted, with an emphasis on the importance of nanoconfinement and interfacial effects. Finally, the challenges and future perspectives of ball-milled Mg-based hydrides for practical on-board hydrogen storage applications are outlined. This review aims to provide valuable insights and guidance for the development of advanced Mg-based hydrogen storage materials with superior performance.

## 1. Introduction

Hydrogen, as a clean and sustainable energy carrier, has attracted increasing attention for its potential to mitigate the environmental and energy crisis. Mg-based materials are promising candidates for solid-state hydrogen storage due to their high theoretical capacity (7.6 wt.% for MgH_2_), low cost, and abundant reserves [1,2,3,4]. However, the high thermodynamic stability and sluggish kinetics of Mg-based hydrides have hindered their practical applications [5,6,7]. To address these challenges, various strategies have been developed, among which ball milling has emerged as a versatile and effective technique for the synthesis and modification of Mg-based hydrogen storage materials [8,9,10].

Ball milling is a solid-state processing technique that involves repeated welding, fracturing, and rewelding of powder particles in a high-energy ball mill [11]. The high-energy impacts and shear forces during milling can induce a variety of physical and chemical changes in the materials, such as reductions in particle size, the introduction of defects, the formation of metastable phases, and enhancements in surface reactivity [12]. These microstructural and morphological changes can significantly improve the hydrogen storage properties of Mg-based materials, including their hydrogen capacity, absorption/desorption kinetics, and cycle stability [13,14]. Mechanical ball milling, as an efficient solid-state reaction method, exhibits many unique advantages in material mixing and mechanical alloying processes. Through the high-speed rotation of the ball milling media, using shear and impact forces, it achieves efficient mixing and reaction of materials, not only enhancing material uniformity but also significantly promoting chemical reactions and alloying processes between materials. This method has strong controllability, allowing precise adjustment of the final product properties by modulating the milling time, speed, and media. Compared with traditional alloying methods, mechanical ball milling does not require an external heat source, significantly saving energy and costs. Moreover, it has a wide applicability and is suitable for processing metals, ceramics, plastics, and complex alloy systems, demonstrating excellent versatility and flexibility. Mechanical ball milling also has good scalability and is suitable for both large-scale industrial production and small-scale experimental research, showing important practical application value.

In recent years, extensive research efforts have been devoted to the ball milling of Mg-based hydrogen storage materials, leading to substantial progress in understanding the underlying mechanisms and structure–property relationships. Various Mg-based hydrides, nanostructures, catalysts, and nanocomposites with enhanced hydrogen storage performance have been synthesized via ball milling [15,16,17]. Moreover, advanced characterization techniques and theoretical calculations have provided deep insights into the microstructural evolution and hydrogenation/dehydrogenation behaviors of the ball-milled materials [18,19].

This review aims to provide a comprehensive summary of the state-of-the-art progress in the ball milling of Mg-based hydrogen storage materials. The synthesis of Mg-based hydrides, nanostructures, and catalysts via ball milling will be systematically reviewed, with an emphasis on the effects of milling parameters and additives on the microstructure and hydrogen storage properties. The mechanisms of hydrogen absorption/desorption in the ball-milled materials will be discussed based on advanced characterization and theoretical studies. The limitations and future perspectives of ball milling in advancing Mg-based hydrogen storage materials towards practical applications will also be outlined.

## 2. Synthesis of Mg-Based Hydrides via Ball Milling

### 2.1. Mg-Based Binary Hydrides

MgH_2_ is an efficient hydrogen storage material with a theoretical hydrogen storage capacity of up to 7.6 wt.%. It can be synthesized through various methods, such as hydrogenation and chemical vapor deposition, producing high-purity MgH_2_. However, its high thermodynamic stability (reaction enthalpy change ΔH = −74.5 kJ·mol^−1^ H_2_, entropy change ΔS = −135 J·K^−1^·mol^−1^ H_2_) results in a high dehydrogenation temperature (>350 °C) and slow reaction rate. The discrepancy between the actual and theoretical hydrogen storage capacity significantly limits its practical application [20]. At present, ball milling has been widely used to synthesize MgH_2_ with improved hydrogen storage properties.

Ball milling under a hydrogen atmosphere has proven to be an effective strategy to mitigate particle agglomeration and oxidation issues that commonly arise during the milling process. Lu et al. [21] reported that after milling, the surface area of pure Mg exhibited a substantial increase from 0.61 m^2^/g to 6.16 m^2^/g, accompanied by a reduction in crystal size. The beneficial effects of milling under a hydrogen atmosphere were further demonstrated by the enhanced hydrogen storage capacity of the milled Mg powder. At 350 °C, the hydrogen storage capacity of Mg powder milled under a hydrogen atmosphere reached 3.36 wt.% H_2_, a significant improvement compared to the 0.14 wt.% achieved using conventional ball milling.

The hydrogen storage properties of ball-milled MgH_2_ can be further improved by optimizing the milling parameters, such as milling time, milling speed, ball-to-powder ratio, and hydrogen pressure [22]. Varin et al. [23] systematically investigated the effects of milling time on the microstructure and hydrogen storage properties of MgH_2_. They found that the hydrogen desorption temperature decreased with increasing milling time, reaching a minimum of 277 °C after 20 h of milling. Prolonged milling led to the agglomeration of MgH_2_ particles and the introduction of impurities, which deteriorated the hydrogen storage properties.

### 2.2. Mg-Based Ternary Hydrides

Ball milling has also been applied to synthesize Mg-based ternary and complex hydrides with improved thermodynamic and kinetic properties. By adding metal elements, the crystal structure can be regulated to promote the formation of new crystal phases, improving hydrogen absorption and desorption kinetics and stability. The interfacial catalytic effects of metal elements accelerate hydrogen absorption and desorption, reducing the activation energy of the reaction. It adjusts the hydrogen diffusion behavior in MgH_2_, speeding up hydrogenation and dehydrogenation reactions, enhancing thermodynamic stability, lowering thermal decomposition temperatures, and extending cycle life. Mg_2_CoH_5_, with a hydrogen content of 4.5 wt.%, has been reported to have a lower decomposition temperature and faster kinetics than MgH_2_ [24]. Apart from Mg_2_CoH_5_, other Mg-based binary hydrides, such as Mg_2_NiH_4_ and Mg_2_FeH_6_, have also been successfully synthesized via ball milling. Mg_2_NiH_4_ exhibits a higher hydrogen content of 5 wt.% and a lower decomposition temperature of 240 °C compared to Mg_2_CoH_5_ [25].

Complex hydrides, such as Mg(NH_2_)_2_ and Mg(BH_4_)_2_, have attracted increasing attention due to their high hydrogen contents (7.2 wt.% for Mg(NH_2_)_2_ and 14.9 wt.% for Mg(BH_4_)_2_) [26,27]. However, their high decomposition temperatures and poor reversibility have hindered their practical applications. Ball milling has been used to synthesize these complex hydrides with improved hydrogen storage properties. The ball-milled Mg(NH_2_)_2_ exhibited a hydrogen desorption temperature of 150 °C, which was 250 °C lower than that of the original Mg(NH_2_)_2_.

Mg(BH_4_)_2_ has been synthesized by ball milling a mixture of MgB_2_ and LiBH_4_ [28]. The ball-milled Mg(BH_4_)_2_ showed a hydrogen desorption temperature of 260 °C, which was 140 °C lower than that of the as-synthesized Mg(BH_4_)_2_. The improved kinetics were attributed to the reduced particle size and the formation of LiMg(BH_4_)_3_ during ball milling, which destabilized the Mg(BH_4_)_2_ structure. Table 1 summarizes the preparation methods and hydrogen storage properties of Mg-based ternary and complex hydrides. Ball milling of MgH_2_ with LiNH_2_ and LiBH_4_ can produce Mg(NH_2_)_2_ and Mg(BH_4_)_2,_ respectively. These complex hydrides have higher hydrogen capacities and lower dehydrogenation temperatures than binary hydrides. Mg(NH_2_)_2_ decomposes to Li_2_Mg(NH)_2_ during dehydrogenation, while ball milling of Mg(BH_4_)_2_ with LiBH_4_ forms LiMg(BH_4_)_3_.

### 2.3. Mechanisms of Mg-Based Hydride Formation during Ball Milling

The formation of Mg-based hydrides during ball milling involves a series of physical and chemical processes, including particle refinement, surface activation, and chemical reactions [29]. The high-energy impacts during ball milling can effectively reduce the particle size and create fresh surfaces, which facilitate the diffusion and dissociation of hydrogen molecules. The accumulation of defects and strain energy during milling also enhances the reactivity of the Mg-based materials towards hydrogen [30].

The formation mechanism of MgH_2_ during ball milling under a hydrogen atmosphere has been extensively studied. Czujko et al. [31] proposed a three-stage mechanism for the formation of MgH_2_ during ball milling: (i) refinement of Mg particles and creation of fresh surfaces; (ii) chemisorption of hydrogen on the Mg surfaces and nucleation of MgH_2_; (iii) growth of MgH_2_ phase and further refinement of the particles. The rate-limiting step is the nucleation of MgH_2_, which requires a critical hydrogen pressure and a sufficient number of active sites on the Mg surfaces.

For Mg-based binary and ternary hydrides, the formation mechanism during ball milling involves the interdiffusion and reaction of the constituent elements under a hydrogen atmosphere. The high-energy impacts can induce the formation of metastable phases and enhance the atomic diffusion, which facilitate the formation of the hydride phases [32]. For example, during the ball milling of Mg and Ni powders under a hydrogen atmosphere, the formation of Mg_2_NiH_4_ involves the following steps: (i) formation of Mg_2_Ni alloy; (ii) hydrogenation of Mg_2_Ni to form Mg_2_NiH_0_._3_; (iii) disproportionation of Mg_2_NiH_0_._3_ into MgH_2_ and Mg_2_NiH_4_.

The formation of Mg-based complex hydrides during ball milling involves the solid-state reaction between MgH_2_ and the complex hydride precursors (e.g., LiNH_2_, LiBH_4_). The high-energy impacts can break the B-H and N-H bonds in the complex hydrides and facilitate the exchange of H atoms with MgH_2_, leading to the formation of Mg(NH_2_)_2_ and Mg(BH_4_)_2_ [33]. The simultaneous refinement of the particles and mixing of the reactants also enhance the contact area and reactivity, which promote the solid-state reactions.

## 3. Nanostructuring of Mg-Based Hydrides via Ball Milling

Nanostructuring has been recognized as an effective strategy to improve the hydrogen storage properties of Mg-based hydrides [34]. Nanostructured materials have a high surface-to-volume ratio, short diffusion paths, and a large number of grain boundaries and defects, which can enhance the hydrogen absorption/desorption kinetics and reduce the thermal stability [35]. Ball milling is a simple and efficient technique to produce nanostructured Mg-based hydrides with a high yield and low cost.

### 3.1. Nanocrystalline Mg-Based Hydrides

The reversible reaction of hydrogen with metal Mg to form MgH_2_ has special properties for energy storage. However, the high thermodynamic stability of the hydride results in high absorption/desorption temperatures. Nanocrystalline Mg-based hydrides have been extensively studied for hydrogen storage applications. Modifying processing routes or combinatorial routes can also lead to refined microstructures, including methods such as melt spinning, cold rolling, and mechanical milling, which can be used to prepare nanocrystalline MgH_2_ structures.

As the particle size is reduced below a critical value, a remarkable decrease in the desorption temperature is observed. Specifically, when the particle size is reduced to the range of 500–600 nm, the onset desorption temperature of MgH_2_ exhibits a substantial reduction of approximately 40–60 °C. Microstructural analysis of MgH_2_ powder subjected to ball milling for durations exceeding 10 h reveals the coexistence of metastable γ-MgH_2_ and stable nanocrystalline β-MgH_2_ phases. Quantitative evidence suggests that the refinement of powder granularity and the presence of the γ-MgH_2_ phase within the particles are the primary factors contributing to the significant reduction in the desorption temperature of MgH_2_ hydride [36].

MgH_2_ nanowires exhibit significantly lower desorption barriers (33.5 and 38.8 kJ mol^−1^ for hydrogenation and dehydrogenation, respectively) compared to commercial MgH_2_ (120–142 kJ·mol^−1^). Table 2 summarizes the preparation conditions and hydrogen storage properties of nanocrystalline Mg-based hydrides. Theoretical predictions suggest that reducing the diameter of the nanowires below 30 nm can substantially influence the thermodynamics and kinetics of MgH_2_ [37]. Furthermore, experimental investigations on the hydrogen storage performance of MgH_2_ with varying particle sizes (25, 32, 38 nm) provide compelling evidence that smaller particle sizes lead to enhanced hydrogenation kinetics [38]. 

### 3.2. Amorphous Mg-Based Hydrides

Amorphous Mg-based hydrides have also been synthesized via ball milling and have shown improved hydrogen storage properties compared to their crystalline counterparts. Unlike crystalline materials, amorphous hydrides lack long-range atomic order and have a high density of defects and free volume, which can facilitate hydrogen diffusion and reduce the stability of the hydrides [39,40,41].

Zhang et al. [42] synthesized amorphous CeMg_11_Ni alloys with varying Ni contents (x wt.% Ni, where x = 100, 200) via ball milling. The degree of amorphization of the alloy was found to increase with increasing Ni contents. The hydrogen storage capacities of the alloys with x = 100 and x = 200 were determined to be 5.94 wt.% and 6.15 wt.%, respectively. Moreover, the dehydrogenation rate exhibited a consistent increase with increasing Ni contents.

Liang et al. [43] successfully synthesized an amorphous La@Mg compound by ball milling a mixture of (La(acac)_3_) and Mg. This composite material exhibited a remarkable hydrogen storage capacity of approximately 7.6% and a hydrogen desorption rate of 7.2%, surpassing the performance of its crystalline La@Mg counterpart and pure Mg. The adsorption/desorption kinetics were rapid, and the reversible adsorption/desorption cycles demonstrated excellent stability. Theoretical calculations in conjunction with experimental results revealed that the amorphous La@Mg structure provides channels for enhanced hydrogen diffusion. This unique feature facilitates the hydrogenation process by accelerating the diffusion of H atoms between the subsurface and surface regions. Table 3 summarizes the preparation conditions and hydrogen storage properties of amorphous Mg-based hydrides. Long-time high-energy ball milling with the addition of alloying elements can produce amorphous MgH_2_ and Mg_2_NiH_4_. Amorphous hydrides exhibit better hydrogen storage kinetics than their crystalline counterparts due to the increased defects and free volume. The addition of TiF_3_ and VTiCr can further improve the amorphization and hydrogen storage properties of MgH_2_ and Mg_2_NiH_4_.

### 3.3. Mechanisms of Nanostructure/Amorphous Formation during Ball Milling

**Figure 1:**
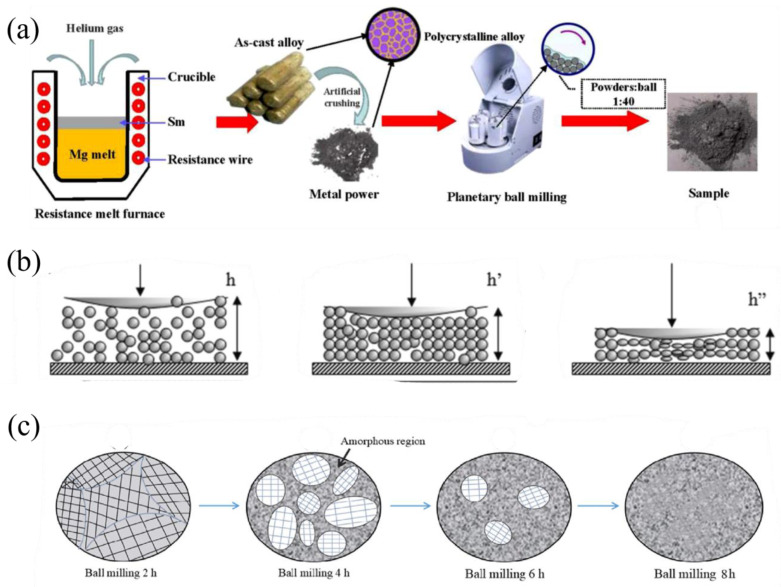
(**a**) Flowchart for the preparation method of the as-milled Sm_5_Mg4_1_ alloy [45]. (**b**) Illustration of the deformation of powder agglomerate during the impact process [46]. (**c**) Illustration of the different microstructural states of the Mg_2_Ni alloys [50].

The formation of nanostructures during ball milling involves a series of physical and chemical processes, including particle refinement, strain accumulation, and phase transformation [44]. Figure 1a illustrates the preparation process, where high-energy collisions during ball milling effectively reduce the particle size and introduce a high density of defects and strain energy into the materials [45].

The formation mechanism of nanocrystalline structures during ball milling has been extensively studied. As depicted in Figure 1b, the high-energy impacts during ball milling lead to repeated particle deformation, fractures, and welding, resulting in a significant reduction in particle size and a concomitant increase in surface area [46]. Fecht [47] proposed a three-stage model for the formation of nanocrystalline structures during ball milling: (i) initial stage: refinement of particles and introduction of defects; (ii) intermediate stage: formation of nanocrystalline domains and grain boundaries; (iii) final stage: further refinement of nanocrystalline domains and saturation of grain size. The final grain size is determined by the balance between the plastic deformation and dynamic recovery during ball milling.

The formation of amorphous structures during ball milling involves the accumulation of strain energy and the suppression of crystallization [48]. When the strain energy stored in the materials reaches a critical value, the crystalline structure becomes unstable and transforms into an amorphous state. The critical strain energy depends on the nature of the materials and the milling conditions. For Mg-based hydrides, the formation of amorphous structures is favored by the presence of additives (e.g., Ni, Y) and the high energy input during ball milling [49]. Figure 1c reveals the evolution of the microstructure during the milling process. As the milling progresses, amorphous phases gradually nucleate and grow at the expense of the crystalline phases due to the accumulation of strain energy. The crystalline domains continuously shrink and ultimately disappear, resulting in a fully amorphous structure [50].

The formation of nanostructures during the ball milling process is influenced by several factors, including the hydrogen pressure and the presence of additives. Cuevas et al. [51] successfully synthesized MgH_2_-TiH_2_ nanocomposites with grain sizes ranging from 4 to 12 nm through reactive ball milling under a hydrogen pressure of 8 MPa. The catalytic properties of TiH_2_ played a crucial role in accelerating the formation of the MgH_2_ phase, enabling the nanocomposite to form within a remarkably short duration of less than 50 min. Furthermore, the TiH_2_ phase effectively inhibited the coarsening of Mg grains, allowing the MgH_2_ phase to nucleate and subsequently form a continuous hydride layer within the Mg nanoparticles.

## 4. Catalytic Modification of Mg-Based Hydrides via Ball Milling

Catalytic modification has been widely used to enhance the hydrogen storage properties of Mg-based hydrides [52]. The addition of catalysts can lower the activation energy for hydrogen absorption/desorption, increase the reaction kinetics, and improve the reversibility and cycling stability of the hydrides [53]. Ball milling is an effective technique to introduce catalysts into Mg-based hydrides and achieve a uniform distribution and strong interaction between the catalysts and the hydrides [54].

### 4.1. Transition Metal Catalysts

Transition metals, such as Ti, V, Mn, Fe, Co, Ni, Cu, and Nb, have been extensively studied as catalysts for Mg-based hydrides [55]. These metals have a high catalytic activity for hydrogen dissociation and recombination and can form stable hydrides with a high hydrogen storage capacity [56]. The catalytic effects of transition metals on the hydrogen storage properties of Mg-based hydrides have been attributed to several mechanisms, including the spillover effect, the gateway effect, and the hydride-forming effect [57,58,59].

Liang et al. [60] investigated the catalytic effects of different transition metals (Ti, V, Mn, Fe, Ni) on the hydrogen storage properties of MgH_2_. They found that the addition of 1 mol% of transition metals via ball milling significantly reduced the dehydrogenation temperature and activation energy of MgH_2_. Composites with Ti or V added exhibit rapid desorption kinetics above 250 °C and rapid adsorption kinetics below 25 °C. The enhanced catalytic effect of Ti and V was attributed to their strong interaction with MgH_2_ and the formation of stable hydrides (TiH_2_ and VH_2_) during dehydrogenation.

Wu et al. [61] facilitated the ball milling of Nb and MgH_2_ using surfactants. The MgH_2_-5 wt.% Nb composite material began releasing hydrogen at 186.7 °C, with a maximum release of 7.0 wt.%. It released 4.2 wt.% H_2_ within 14 min at 250 °C and could absorb 4.0 wt.% H_2_ within 30 min, even at the low temperature of 100 °C. The desorption activation energy and hydrogenation activation energy were reduced from 140.51 ± 4.74 and 70.67 ± 2.07 kJ·mol^−1^ to 90.04 ± 2.83 and 53.46 ± 3.33 kJ·mol^−1^, respectively.

The catalytic effects of transition metals on the hydrogen storage properties of Mg-based hydrides can be further enhanced by optimizing the ball milling conditions and the catalyst composition. Lu et al. [62] successfully synthesized a MgH_2_-0.1TiH_2_ nanocomposite system with exceptionally fine nanocrystalline grain sizes ranging from 5 to 10 nm. This was achieved through an innovative ultra-high-energy-high-pressure mechanical milling approach. The TiH_2_ phase was homogeneously dispersed among the MgH_2_ particles, forming a well-distributed nanocomposite. The synergistic effects of the nanoscale structure and the catalytic influence of TiH_2_ led to a remarkable enhancement in the dehydrogenation and hydrogenation kinetics of the MgH_2_-0.1TiH_2_ system compared to commercial MgH_2_.

Cui et al. [63] developed an innovative approach to enhance the catalytic effects of Ti on MgH_2_ by coating ball-milled Mg powder (approx. 1 μm in diameter) with a multivalent Ti-based catalyst. The coating process involved a chemical reaction between the Mg powder and TiCl_3_ in a THF solution, resulting in the formation of a 10 nm thick catalyst layer. This layer contained multiple valence states of Ti, including Ti, TiH_2_, TiCl_3_, and TiO_2_. The presence of these diverse Ti valence states played a crucial role in facilitating electron transfer between Mg^2+^ and H^−^, thereby promoting the recombination of H_2_ on the Ti surface. The resulting MgH_2_-encapsulated Ti-based system (Mg-Ti) demonstrated a remarkable dehydrogenation performance, with hydrogen release initiating at around 175 °C and achieving a release of 5 wt.% H_2_ within a mere 15 min at 250 °C.

Table 4 summarizes the effects of transition metal catalysts on the hydrogen storage properties of Mg-based hydrides. The addition of Ti, V, Mn, Fe, Ni, Nb, and their alloys via ball milling can significantly improve the hydrogen storage kinetics of MgH_2_ and Mg_2_NiH_4_. Ti and V show the best catalytic effects, while the catalytic effects of Mn, Fe, and Ni are relatively weak. The catalytic effects depend on the catalyst composition, milling time, and milling parameters.

### 4.2. Metal Oxide Catalysts

Metal oxides such as Nb_2_O_5_, TiO_2_, V_2_O_5_, Cr_2_O_3_, and Al_2_O_3_ have also been studied as catalysts for Mg-based hydrides [64]. These oxides have a high chemical stability and a strong interaction with hydrides, and they can act as dispersants and grain growth inhibitors during ball milling [65]. The catalytic effects of metal oxides on the hydrogen storage properties of Mg-based hydrides have been attributed to several mechanisms, including the formation of active species, the creation of hydrogen diffusion channels, and the modification of the electronic structure of the hydrides [66].

Barkhordarian et al. [67] investigated the catalytic effects of Nb_2_O_5_ on the hydrogen storage properties of MgH_2_. By ball milling 0.5 mol.% Nb_2_O_5_ with MgH_2_, they observed a significant enhancement in the reaction kinetics of the composite. The catalytic influence of Nb_2_O_5_ was particularly evident at elevated temperatures. At 300 °C, the composite exhibited rapid hydrogen release, achieving a 7 wt.% H_2_ release within a mere 90 s. Similarly, the composite demonstrated fast hydrogen absorption, with 7 wt.% H_2_ being absorbed within just 60 s at the same temperature. Even at a lower temperature of 250 °C, the composite showcased impressive kinetics, absorbing over 6 wt.% H_2_ within 60 s and subsequently releasing it within 500 s. 

Polanski et al. [68] explored the catalytic effects of nanocrystalline Cr_2_O_3_ on the hydrogen storage properties of MgH_2_. Interestingly, they discovered that the addition of Cr_2_O_3_ did not significantly influence the grain size of β-MgH_2_. However, despite the lack of grain size reduction, the presence of Cr_2_O_3_ had a profound impact on the reaction kinetics of the composite. The MgH_2_-Cr_2_O_3_ composite exhibited remarkable hydrogen absorption and desorption rates. At 300 °C and a hydrogen pressure of 1 MPa, the composite could absorb 6 wt.% H_2_ within an impressively short duration of 2 min. Moreover, under the same temperature conditions, the composite demonstrated rapid hydrogen release, achieving a 6 wt.% H_2_ release within just 10 min.

The catalytic effects of metal oxides on the hydrogen storage properties of Mg-based hydrides can be further enhanced by optimizing the ball milling conditions and the catalyst morphology. Ma et al. [69] conducted a comprehensive study on the influence of catalyst morphology on the hydrogen storage properties of MgH_2_. They discovered that the morphology of the catalyst played a crucial role in determining its surface energy and chemical interactions with Mg and hydrogen. To investigate this effect, they synthesized anatase TiO_2_ with different crystal face advantages using a hydrothermal method. These TiO_2_ catalysts, denoted as TF_x_ (x = 0, 10, 30, 50, 70, and 80), were then incorporated into MgH_2_ via ball milling. Among the prepared composites, MgH_2_-TF_70_ exhibited the most promising hydrogen adsorption kinetics, with an exceptionally low apparent activation energy for dehydrogenation of only 76.1 ± 1.6 kJ mol^−1^. This composite material demonstrated an initial hydrogen release temperature of approximately 220 °C. Furthermore, it showcased rapid hydrogen absorption and desorption capabilities, absorbing 5.3 wt.% H_2_ within a remarkable 44 s at 200 °C and releasing 6.4 wt.% H_2_ within 700 s at 300 °C.

Table 5 summarizes the effects of metal oxide catalysts on the hydrogen storage properties of Mg-based hydrides. The addition of Nb_2_O_5_, TiO_2_, Cr_2_O_3_, and V_2_O_5_ via ball milling can improve the hydrogen storage kinetics of MgH_2_. The catalytic effects of metal oxides are relatively weaker than those of transition metals, but they can be enhanced by optimizing the milling conditions. The milling atmosphere, milling time, and milling parameters have significant effects on the catalytic activity and stability of metal oxides.

### 4.3. Mechanisms of Catalytic Effects during Ball Milling

The catalytic effects of transition metals and metal oxides on the hydrogen storage properties of Mg-based hydrides involve a series of physical and chemical processes during ball milling and hydrogen absorption/desorption cycles [70]. The high-energy impacts during ball milling can induce the refinement and uniform distribution of the catalysts in the hydride matrix, creating a large number of catalyst–hydride interfaces and active sites for hydrogen storage [71]. The catalysts can also act as dispersants and grain growth inhibitors, preventing the agglomeration and coarsening of the hydride particles during prolonged milling and cycling [72].

During the hydrogen absorption/desorption processes, the catalysts can facilitate the dissociation and recombination of hydrogen molecules on the surface of the hydrides, lowering the activation energy and increasing the reaction rates [73]. The dissociated hydrogen atoms can migrate from the catalyst surface to the hydride surface via the spillover effect and then diffuse into the bulk of the hydride via the gateway effect [74]. The presence of catalysts can also modify the electronic structure of the hydrides, weakening the Mg-H bonds and facilitating hydrogen desorption [75].

The specific catalytic mechanisms depend on the nature of the catalysts and the hydrides, as well as the reaction conditions. For transition metal catalysts, the formation of stable hydrides (e.g., TiH_2_, VH_2_, NbH_0_._89_) during the hydrogen absorption/desorption cycles can provide additional catalytic sites and hydrogen diffusion channels, enhancing the overall kinetics [76]. For metal oxide catalysts, the reduction of the oxides to lower valence states or metallic phases during the hydrogen absorption/desorption cycles can create active species and defects, which facilitate the hydrogen storage reactions [77].

The catalytic mechanisms can also involve the formation of intermediate phases and solid solutions during the hydrogen absorption/desorption cycles. For example, the addition of Fe to MgH_2_ can lead to the formation of the Mg_2_FeH_6_ intermediate phase during hydrogen absorption, which enhances the hydrogen diffusion and storage capacity [78]. The addition of Nb to MgH_2_ can lead to the formation of a Mg-Nb solid solution during dehydrogenation, which improves the reversibility and cycling stability of the hydride [79].

**Figure 2:**
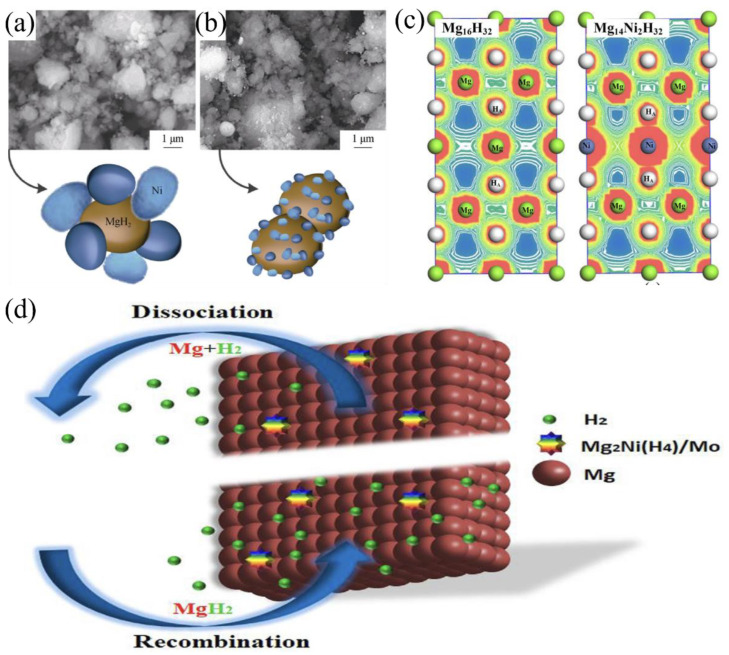
SEM images and schematic models of samples: (**a**) MgH_2_-mNi (2 h); (**b**) MgH_2_-nNi (2 h) [80]; (**c**) total charge densities in (110) crystal planes of Mg_16_H_32_,Mg_14_Ni_2_H_32_ [81]; (**d**) schematic diagram of the de/re-hydrogenation processes of the MgH_2_ + NiO@NiMoO_4_ composite [82].

To elucidate the catalytic mechanisms of transition metals, we take Ni as a representative example. Figure 2a presents micron-sized Ni-encapsulated MgH_2_ composite materials with an average particle size ranging from 2 to 10 μm, where the Ni particles exhibit a size comparable to that of MgH_2_ [80]. In contrast, Figure 2b showcases MgH_2_-nNi (2 h) samples, revealing nanoscale Ni particles encapsulating MgH_2_ with an average particle size of 1–5 μm. Compared to pure MgH_2_, the incorporation of Ni via doping leads to the partial substitution of Mg atoms, resulting in an increased overlap of electron clouds (Figure 2c) [81]. This phenomenon indicates the formation of stronger covalent interactions between Mg and Ni atoms, which consequently weakens the Mg-H bond strength. As a result, the kinetics of hydrogen absorption and desorption reactions in the Ni-doped MgH_2_ composite material are significantly enhanced [81].

In the case of metal oxides, the in situ formation of catalytically active alloy phases during the hydrogen absorption and desorption processes plays a pivotal role in enhancing the hydrogen storage performance of Mg-based hydrides. Figure 2d illustrates the catalytic mechanism of NiO/NiMoO_4_-doped MgH_2_ composite materials. During the hydrogen absorption and desorption processes, the composite undergoes an in situ formation of catalytically active Mg_2_Ni/Mg_2_NiH_4_ and Mo phases. These newly formed species play a crucial role in accelerating the dissociation of H_2_ molecules on the surface of MgH_2_. Moreover, they create a “hydrogen pump” effect, facilitating the reversible hydrogen absorption and desorption in MgH_2_ under relatively mild conditions [82].

The optimization of the catalytic effects requires a comprehensive understanding of the structure–property relationships and the reaction mechanisms of the catalyst–hydride systems. Advanced characterization techniques such as X-ray diffraction (XRD), transmission electron microscopy (TEM), X-ray photoelectron spectroscopy (XPS), and neutron scattering have been used to investigate the microstructure, morphology, electronic structure, and hydrogen storage properties of ball-milled Mg-based hydrides with catalysts. Theoretical calculations such as density functional theory (DFT) and molecular dynamics (MD) simulations have also been used to predict the catalytic activity and the hydrogen storage mechanisms of the catalyst–hydride systems.

## 5. Nanocomposite Mg-Based Hydrides via Ball Milling

Nanocomposite Mg-based hydrides, consisting of nanosized Mg-based hydrides and other functional materials (e.g., carbon materials, metal hydrides, metal organic frameworks), have attracted increasing attention for hydrogen storage applications [83]. The incorporation of functional materials into the Mg-based hydrides can create synergistic effects, such as improved thermal conductivity, enhanced hydrogen diffusion, and reduced hydride stability, leading to superior hydrogen storage properties [84]. Ball milling is a simple and effective technique to prepare nanocomposite Mg-based hydrides with a uniform distribution and strong interaction between the components.

### 5.1. Carbon-Containing Nanocomposites

Carbon materials, such as graphite, carbon nanotubes (CNTs), and graphene, have been widely used to prepare nanocomposite Mg-based hydrides due to their high thermal conductivity, high surface area, and excellent mechanical properties [85]. The addition of carbon materials to Mg-based hydrides via ball milling can enhance the thermal management and hydrogen diffusion of the system, leading to improved hydrogen absorption/desorption kinetics and reversibility [86].

Pal et al. [87] reported a significant reduction in the dehydrogenation temperature of MgH_2_ by ball milling with 10 wt.% flake graphene. The dehydrogenation temperature decreased from 410 °C for pure MgH_2_ to 282 °C for the MgH_2_–graphene composite. This reduction in temperature is noteworthy, as it is substantially lower than the dehydrogenation temperature of pure MgH_2_. The incorporation of graphene also led to a decrease in the dehydrogenation activation energy from 170 kJ/mol for pure MgH_2_ to 136 ± 2 kJ/mol for the composite. Interestingly, XRD analysis before and after the dehydrogenation reaction confirmed the presence of graphene, indicating that no direct chemical reaction occurred between graphene and MgH_2_.

Rather et al. [88] investigated the hydrogen storage properties of ball-milled MgH_2_ with 5 wt.% carbon nanotubes (CNTs) and observed a hydrogen absorption capacity of 5.5 wt.% under a hydrogen pressure of 4.6 MPa at 673 K. Kinetic rate modeling studies provided valuable insights into the role of CNTs in enhancing hydrogen storage performance. The results indicated that CNTs facilitate the diffusion of hydrogen within the Mg matrix, thereby accelerating the formation of the hydride phase. Furthermore, the interaction between Mg and C atoms created abundant sites for hydrogen dissociation and diffusion. The unique tubular structure of CNTs also contributed to improved internal H_2_ transport within the composite material.

Peng et al. [89] synthesized a novel MgH_2_@CA microsphere composite material by ball milling carbon aerogel (CA) microspheres, which possessed a mesh-like internal structure, with Mg powder followed by an activation process. The resulting composite exhibited a distinctive core–shell structure that significantly enhanced the hydrogenation and dehydrogenation rates. The composite material demonstrated rapid hydrogen absorption, achieving a capacity of 6.2 wt.% H_2_ within a mere 5 min at 275 °C. Additionally, it exhibited fast hydrogen release, liberating 4.9 wt.% H_2_ within 100 min at 350 °C. The apparent activation energy for dehydrogenation was remarkably reduced to 114.8 kJ/mol. These improvements were attributed to the in situ-formed MgH_2_ nanoparticles, which reduced the diffusion distance of H_2_, and the CA matrix, which provided nucleation sites and prevented particle agglomeration.

Table 6 summarizes the effects of carbon materials on the hydrogen storage properties of Mg-based hydride nanocomposites. The addition of graphite, carbon nanotubes (CNTs), graphene, and fullerene (C60) via ball milling can significantly improve the hydrogen storage kinetics of MgH_2_ and Mg_2_NiH_4_. The catalytic effects of carbon materials depend on their morphology, size, structure, and content. Carbon materials can enhance the thermal conductivity, facilitate the hydrogen diffusion, and prevent the particle aggregation of Mg-based hydrides.

### 5.2. Metal Hydride-Containing Nanocomposites

Metal hydrides such as LiBH_4_, NaAlH_4_, and TiH_2_ have been used to prepare nanocomposite Mg-based hydrides due to their high hydrogen storage capacity and catalytic effects [90,91]. The addition of metal hydrides to Mg-based hydrides via ball milling can create hydrogen diffusion channels and active sites, leading to enhanced hydrogen absorption/desorption kinetics and reversibility [92].

**Figure 3:**
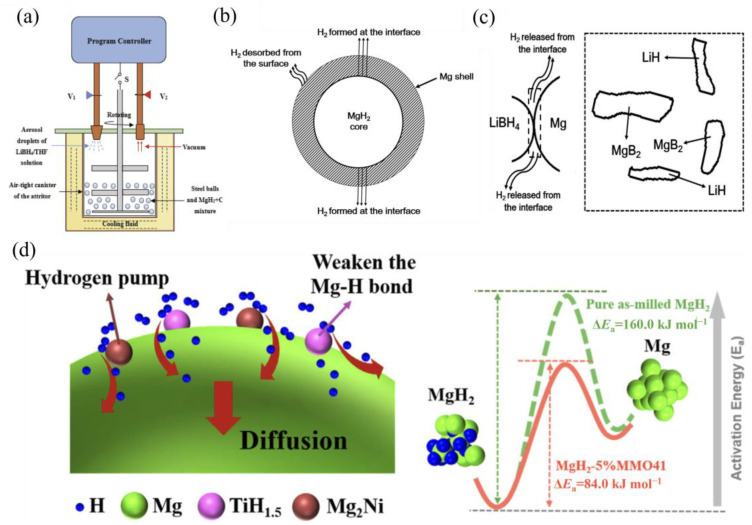
(**a**) Schematic of the automated ball milling with an aerosol spraying device. (**b**) Schematic of the dehydrogenation via the MgH_2_-Mg pathway. (**c**) The reaction takes place at the Mg/LiBH_4_ interface, leading to the nucleation and growth of MgB_2_ + LiH products (shown inside the dashed box) [93]. (**d**) Schematic illustration of the synergistic effects of the TiH_1.5_−Mg_2_Ni nanocatalyst [96].

Ding et al. [93] further advanced the ball milling approach by introducing LiBH_4_ nanoparticles in aerosol form (Figure 3a), which resulted in the formation of MgH_2_-LiBH_4_ composite materials with a superior hydrogen release performance. Figure 3b reveals the hydrogen release mechanism in pristine MgH_2_, where the contraction of the MgH_2_ core during dehydrogenation leads to the formation of a continuous Mg shell. This Mg shell acts as a barrier, hindering the decomposition of MgH_2_ and the diffusion of H_2_ molecules. However, the introduction of LiBH_4_ nanoparticles alters the reaction pathway (Figure 3c). At the Mg/LiBH_4_ interface, new reactions take place, resulting in the formation of MgB_2_ and LiH. These interfacial reactions effectively break the Mg shell surrounding MgH_2_, thus promoting the release of H_2_.

Kwak et al. [94] employed reactive ball milling to synthesize MgH_2_-NaAlH_4_ composite materials with varying ratios. As the NaAlH_4_ content increased, a notable trend was observed in the curve depicting the ratio of hydrogen release at the temperature peak to the increase in temperature. The composite with the composition MgH_2_-30NaAlH_4_ demonstrated the most promising performance among the prepared samples. This composite exhibited a remarkable hydrogen absorption capacity of 7.42 wt.% within a short duration of 10 min at 320 °C. Moreover, it showcased rapid hydrogen release, liberating the same amount of H within an impressive timeframe of just 20 min at the same temperature.

El-Eskandarany et al. [95] successfully synthesized MgH_2_-5.3 wt.%TiH_2_ composite material via reactive ball milling. The composite exhibited impressive hydrogen storage properties at relatively low temperatures. At 175 °C, the material adsorbed 5.5 wt.% H_2_ within a short duration of 6 min. Similarly, at 250 °C, it released the same amount of H_2_ within just 7 min. Furthermore, the synthesized nanocomposite powder demonstrated remarkable cyclic stability, maintaining its performance for an extended cycle life of up to 580 h without any observable decay.

The improved hydrogen storage properties were attributed to the catalytic effects of the Ti phase formed during the dehydrogenation of TiH_2_, which acted as a gateway for hydrogen diffusion and a seed for the nucleation of MgH_2_. Figure 3d illustrates the catalytic principle of TiH_2_ in the Mg_2_NiH_4_ system. The in situ-formed Ti-H species interact synergistically with Mg_2_Ni, creating additional active sites for hydrogen dissociation and recombination on the MgH_2_ surface. Moreover, these Ti-H species facilitate the diffusion and migration of atomic and molecular hydrogen through a “hydrogen pump” effect, which further enhances the hydrogen absorption and desorption kinetics [96].

Table 7 summarizes the effects of various metal hydrides on the hydrogen storage properties of Mg-based hydride nanocomposites. The addition of LiBH_4_, NaAlH_4_, TiH_2_, CaH_2_, and LaNi_5_ via ball milling can improve the hydrogen storage kinetics and thermodynamics of MgH_2_ and Mg_2_NiH_4_. The formation of reactive hydride composites, the catalytic effects, and the nanoconfinement effects are the main mechanisms for the enhanced hydrogen storage performance. However, the optimal design of the metal hydride additives still needs to be tailored for specific Mg-based hydride systems to achieve the best hydrogen storage properties.

### 5.3. Metal–Organic Framework-Containing Nanocomposites

**Figure 4:**
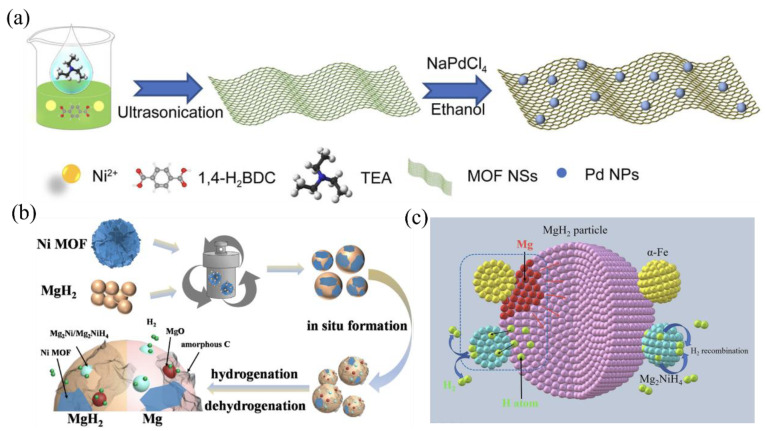
(**a**) Schematic diagram for the preparation of 2D MOF@Pd hybrid nanosheets [99]. (**b**) Schematic illustration of the catalytic mechanism of MgH_2_-5 wt.% Ni MOF [100]. (**c**) Schematic illustration of catalytic mechanism of the MgH_2_-TM MOF (TM = Fe, Ni) composite [101].

Metal–organic frameworks (MOFs), consisting of metal ions and organic linkers, have been used to prepare nanocomposite Mg-based hydrides due to their high surface area, tunable pore size, and functionalized pore surface [97]. The incorporation of MOFs into Mg-based hydrides via ball milling can create hydrogen diffusion channels and active sites, as well as confine the hydride particles within the pores, leading to enhanced hydrogen storage properties [98]. The catalytic effects on the hydrogen absorption and desorption reactions of MgH_2_ can be further enhanced by strategically loading metal nanoparticles onto the surface of MOFs. Figure 4a presents a facile and efficient ultrasonic-assisted approach for the synthesis of Ni-MOF@Pd NP hybrid nanosheets at room temperature. This novel nanostructure combines the high surface area and porosity of MOFs with the catalytic activity of metal nanoparticles, providing a synergistic effect for improved hydrogen storage performance [99].

Gao et al. [100] successfully synthesized a novel flower-shaped Ni-MOF catalyst with enhanced thermal stability (Figure 4b). This Ni-MOF catalyst demonstrated remarkable catalytic activity towards improving the hydrogen storage performance of MgH_2_. The incorporation of 5 wt.% Ni-MOF into MgH_2_ significantly reduces the initial hydrogen release temperature by 73 °C, from 380 °C for pure MgH_2_ to 307 °C for the composite material. Moreover, the Ni-MOF-doped MgH_2_ composite exhibits enhanced dehydrogenation kinetics at 300 °C, achieving a hydrogen release capacity of 6.4 wt.% within a mere 600 s. Remarkably, even after dehydrogenation, the composite material demonstrates the ability to absorb approximately 5.7 wt.% H_2_ at temperatures as low as 150 °C under a hydrogen pressure of 3 MPa.

Ma et al. [101] employed a ball milling approach to combine 10% Fe/Ni bimetallic MOF with MgH_2_, which resulted in a significant reduction in the hydrogen release temperature from 412 °C to 273.9 °C for the composite material. Moreover, this strategic combination of Fe/Ni bimetallic MOF and MgH_2_ led to a notable decrease in the activation energy of the hydrogen release reaction by 45.3 kJ/mol. Figure 4c elucidates the catalytic mechanism of the Fe/Ni bimetallic MOF in the MgH_2_ system. The in situ formation of catalytically active α-Fe and Mg_2_NiH_4_ phases during the dehydrogenation process creates additional active sites on the MgH_2_ surface, which accelerate the dissociation and recombination of H_2_. Simultaneously, the nanoparticles dispersed in the composite material generate a “hydrogen pump” effect, further promoting the diffusion and migration of atomic and molecular hydrogen.

### 5.4. Mechanisms of Nanocomposite Formation and Synergistic Effects

The formation of nanocomposite Mg-based hydrides during ball milling involves the physical mixing and chemical bonding of the Mg-based hydrides and functional materials [102]. The high-energy impacts during ball milling can effectively refine the particle size, increase the surface area, and create intimate contact between the components [103]. The functional materials can also act as dispersants and grinding aids, preventing the agglomeration and cold welding of the Mg-based hydride particles during prolonged milling [104].

The synergistic effects of the nanocomposite Mg-based hydrides on the hydrogen storage properties arise from the interfacial interactions and the confinement effects between the components [105]. The functional materials can provide hydrogen diffusion channels, active sites, and catalytic effects, facilitating the hydrogen absorption/desorption kinetics and the reversibility of the Mg-based hydrides [106]. The nanoconfinement of the Mg-based hydrides within the pores or layers of the functional materials can also prevent their sintering and coarsening during cycling, maintaining the high surface area and the fast kinetics [107].

The specific synergistic mechanisms depend on the nature of the functional materials and the Mg-based hydrides, as well as the ball milling conditions. For carbon-containing nanocomposites, the high thermal conductivity and the catalytic effects of the carbon materials can enhance the heat transfer and the hydrogen diffusion during the hydrogen absorption/desorption processes, while the nanoconfinement of the Mg-based hydrides within the carbon matrix can stabilize their nanostructure and prevent their degradation [108].

For metal hydride-containing nanocomposites, the catalytic effects of the metal hydrides and their decomposition products (e.g., Ti, Al) can promote the dissociation and recombination of hydrogen molecules on the surface of the Mg-based hydrides, while the formation of reactive hydride composites can destabilize the Mg-based hydrides and lower their dehydrogenation temperature and enthalpy [109].

For MOF-containing nanocomposites, the high surface area and the functionalized pore surface of the MOFs can provide more active sites and hydrogen diffusion channels, while the nanoconfinement of the Mg-based hydrides within the MOF pores can prevent their agglomeration and growth during cycling [110]. The catalytic effects of the metal ions and the organic linkers in the MOFs can also facilitate hydrogen absorption/desorption reactions and improve the kinetics and reversibility of Mg-based hydrides [111].

The optimization of nanocomposite Mg-based hydrides requires careful control of the composition, microstructure, and interfacial interactions of the components. Advanced characterization techniques such as XRD, TEM, XPS, and Raman spectroscopy have been used to investigate the phase composition, morphology, electronic structure, and bonding state of the nanocomposite Mg-based hydrides. Theoretical calculations such as DFT and MD simulations have also been employed to predict the hydrogen storage properties and the synergistic mechanisms of nanocomposite Mg-based hydrides.

## 6. Conclusions and Perspectives

Mechanical ball milling has demonstrated significant advantages in the development and preparation of MgH_2_ hydrogen storage materials. Its efficient energy transfer and uniform mixing capabilities promote the homogenization and nanostructuring of MgH_2_, effectively enhancing the material’s hydrogen absorption and desorption performance and reaction rates. By optimizing the milling parameters, mechanical ball milling can significantly reduce the reaction temperature of MgH_2_ and increase its hydrogen storage capacity. Moreover, this method does not require an external heat source, saving energy costs, and it is suitable for both large-scale industrial production and small-scale experimental research. To date, mechanical ball milling technology has achieved numerous breakthroughs in the preparation of MgH_2_ hydrogen storage materials, significantly improving hydrogen storage efficiency and cycle stability and providing a solid foundation and broad application prospects for the development of hydrogen storage technology. Despite the significant progress in the development of ball-milled Mg-based hydrides for hydrogen storage applications, there are still several challenges and issues that need to be addressed for their practical use:

(1) The hydrogen storage capacity of Mg-based hydrides is still lower than the theoretical value due to the presence of impurities, oxides, and by-products introduced during the ball milling process and the hydrogen absorption/desorption cycles. The development of high-purity starting materials, optimized ball milling conditions, and effective purification methods is necessary to maximize the hydrogen storage capacity of Mg-based hydrides.
(2) The hydrogen absorption/desorption kinetics of Mg-based hydrides at low temperatures (<100 °C) is still not satisfactory for practical applications, especially for on-board hydrogen storage in fuel cell vehicles. The development of novel catalysts, nanostructures, and nanocomposites with enhanced low-temperature kinetics is crucial to meet the requirements of practical hydrogen storage systems.
(3) The cyclic stability and reversibility of Mg-based hydrides are still limited by the sintering, coarsening, and degradation of the nanostructure during extended cycling. The development of advanced nanoconfinement and nanoencapsulation strategies, as well as the introduction of anti-sintering additives and coatings, is important to improve the long-term stability and reversibility of Mg-based hydrides.
(4) The safety and compatibility of Mg-based hydrides with the container materials and the fuel cell components are still not well understood and may pose risks for practical applications. The development of advanced characterization techniques and testing protocols, as well as the investigation of the interactions between Mg-based hydrides and other materials, is necessary to ensure the safe and reliable operation of Mg-based hydrogen storage systems.
(5) The cost and scalability of ball milling processes for the production of Mg-based hydrides are still not competitive with other hydrogen storage methods such as compression and liquefaction. The development of low-cost and high-efficiency ball milling techniques, as well as the optimization of the process parameters and the energy consumption, is important to reduce the cost and increase the throughput of Mg-based hydrides.

To address these challenges and advance the development of ball-milled Mg-based hydrides for practical hydrogen storage applications, future research should focus on the following aspects:

(1) The development of novel Mg-based alloys and composites with a high hydrogen storage capacity, fast kinetics, and good reversibility. The use of machine learning and high-throughput screening methods, combined with experimental validation and optimization, can accelerate the discovery and design of new Mg-based hydride materials.
(2) The development of advanced ball milling techniques and equipment for the synthesis and modification of Mg-based hydrides. The use of high-energy and high-frequency ball milling methods, such as planetary ball milling and attritor ball milling, as well as the in situ monitoring and control of the ball milling process, can improve the efficiency and reproducibility of the ball milling process.
(3) The development of multi-scale characterization and modeling tools for the understanding of the structure–property relationships and the hydrogen storage mechanisms of ball-milled Mg-based hydrides. The combination of experimental techniques such as in situ XRD, TEM, and neutron scattering with theoretical methods such as DFT, MD, and phase-field modeling can provide a comprehensive and predictive understanding of the hydrogen storage behavior of Mg-based hydrides.
(4) The development of advanced nanoconfinement and catalysis strategies for the enhancement of the hydrogen storage properties of Mg-based hydrides. The use of novel nanoporous materials, such as MOFs, covalent organic frameworks (COFs), and porous organic polymers (POPs), as well as the functionalization and doping of the catalyst nanoparticles, can create new opportunities for the design and optimization of high-performance Mg-based hydrides.
(5) The development of prototype Mg-based hydrogen storage systems and their integration with fuel cells and other hydrogen utilization technologies. The demonstration and testing of Mg-based hydrogen storage systems under realistic operating conditions, as well as the assessment of their performance, durability, and safety, can provide valuable feedback and guidance for the further improvement and scale-up of Mg-based hydrides.

In conclusion, ball milling has shown great potential for the synthesis and modification of Mg-based hydrides with enhanced hydrogen storage properties. The progress in the understanding of the ball milling mechanisms, the development of advanced characterization and modeling tools, and innovation in the nanostructuring and catalysis strategies have led to significant breakthroughs in the performance of ball-milled Mg-based hydrides. However, there are still challenges and opportunities in the development of practical Mg-based hydrogen storage systems, which require the concerted efforts of researchers from multiple disciplines and collaborations between academia and industry. With continuous research and development, it is expected that ball-milled Mg-based hydrides will play an important role in the transition to a hydrogen-based energy economy and contribute to the sustainable development of our society.

## Figures and Tables

**Table 1:** Preparation methods and hydrogen storage properties of Mg-based ternary and complex hydrides [24,25,26,27,28].

Hydride	Preparation Method	Milling Time (h)	Milling Speed (rpm)	Ball-to-Powder Ratio	Hydrogen Pressure (MPa)	Dehydrogenation Temperature (°C)	Hydrogen Capacity (wt.%)	Desorption Activation Energy (kJ/mol)
Mg_2_CoH_5_	Ball milling of MgH_2_ and Co	2	400	30:1	3.0	265	4.2	121
Mg_2_NiH_4_	Ball milling of MgH_2_ and Mg_2_Ni	3	\	30:1	1.0	240	5	\
Mg(NH_2_)_2_	Ball milling of MgH_2_ and LiNH_2_	3	300	20:1	-	150	7.2	76
Mg(BH_4_)_2_	Ball milling of MgB_2_ and LiBH_4_	1	200	10:1	0.1	260	14.9	118
Li_2_Mg(NH)_2_	Decomposition of Mg(NH_2_)_2_	-	-	-	-	150	5.6	54
LiMg(BH_4_)_3_	Ball milling of Mg(BH_4_)_2_ and LiBH_4_	2	250	15:1	0.1	180	11.5	92

**Table 2:** Preparation conditions and hydrogen storage properties of nanocrystalline Mg-based hydrides [36,37,38].

Hydride	Composition	Milling Time (h)	Milling Speed (rpm)	Ball-to-Powder ratio	Hydrogen Pressure (MPa)	Grain Size (nm)	Dehydrogenation Temperature (°C)	Activation Energy (kJ/mol)	Hydrogen Capacity (wt.%)	Reversible Capacity (wt.%)
MgH_2_	MgH_2_	20	400	10:1	1.0	5–10	200	76	7.2	6.8
Mg_2_NiH_4_	Mg_2_Ni + MgH_2_	25	300	20:1	3.0	3–5	200	87	3.0	2.8
MgH_2_	MgH_2_ + 10 wt.% LiH	1	200	10:1	1.0	7	150	65	6.0	5.8
MgH_2_	MgH_2_ + 5 wt.% Nb_2_O_5_	20	400	30:1	1.0	5–10	200	61	6.5	6.2
Mg_2_NiH_4_	Mg_2_Ni + 10 wt.% TiH_2_	30	250	40:1	3.0	10–20	220	85	3.5	3.2

**Table 3:** Preparation conditions and hydrogen storage properties of amorphous Mg-based hydrides [39,40,41,42,43].

Hydride	Composition	Milling Time (h)	Milling Speed (rpm)	Ball-to-Powder Ratio	Hydrogen Pressure (MPa)	Dehydrogenation Temperature (°C)	Dehydrogenation Activation Energy (kJ/mol)	Hydrogen Capacity (wt.%)	Reversible Capacity (wt.%)
MgH_2_	MgH_2_ + 10 wt.% Ni	20	400	30:1	1.0	150	72	6.8	6.5
Mg_2_NiH_4_	Mg_2_Ni + MgH_2_	100	200	50:1	3.0	200	98	3.0	2.8
Mg-Ni-Y	Mg_65_Ni_30_Y_5_	20	300	40:1	3.0	200	105	3.5	3.2
Mg_2_NiH_4_	Mg_2_Ni + 5 wt.% TiF_3_	50	250	60:1	3.0	180	84	3.2	3.0
MgH_2_	MgH_2_ + 10 wt.% VTiCr	10	500	20:1	1.0	120	63	6.0	5.5

**Table 4:** Effects of transition metal catalysts on the hydrogen storage properties of Mg-based hydrides [56,57,58,59,60,61,62,63].

Hydride	Catalyst (mol%)	Milling Time (h)	Milling Speed (rpm)	Ball-to-Powder Ratio	Dehydrogenation Temperature (°C)	Activation Energy (kJ/mol)	Hydrogen Capacity (wt.%)	Reversible Capacity (wt.%)
MgH_2_	1% Ti, V, Mn, Fe, Ni	1	400	10:1	250 (Ti, V), 300 (Mn, Fe, Ni)	61 (Ti, V), 92 (Mn, Fe, Ni)	6.5 (Ti, V), 6.0 (Mn, Fe, Ni)	6.2 (Ti, V), 5.8 (Mn, Fe, Ni)
MgH_2_	1% Nb	2	500	20:1	200	61	6.8	6.5
MgH_2_	10% Ni	0.5–5	300	30:1	250 (5 h)	67 (5 h)	6.2 (5 h)	6.0 (5 h)
MgH_2_	Ti-Fe-Nb (1:1:1)	2	400	40:1	150	53	6.5	6.2
MgH_2_	5% VTiCr	10	500	20:1	180	59	6.0	5.8
Mg_2_NiH_4_	10% TiH_2_	30	250	60:1	220	85	3.5	3.2

**Table 5:** Effects of metal oxide catalysts on the hydrogen storage properties of Mg-based hydrides [65,66,67,68,69].

Hydride	Catalyst (mol%)	Milling Atmosphere	Milling Time (h)	Milling Speed (rpm)	Ball-to-Powder Ratio	Dehydrogenation Temperature (°C)	Activation Energy (kJ/mol)	Hydrogen Capacity (wt.%)	Reversible Capacity (wt.%)
Mgh_2_	0.5% Nb_2_O_5_	Ar	20	400	30:1	250	85	6.5	6.2
MgH_2_	1% TiO_2_ nanoparticles	Ar	10	500	20:1	275	96	6.2	6.0
MgH_2_	1% Cr_2_O_3_	H_2_	5	400	40:1	225	75	6.8	6.5
MgH_2_	TiO_2_ nanotubes (5%)	Ar	20	300	50:1	250	81	6.5	6.2
MgH_2_	2% Nb_2_O_5_	H_2_	10	500	20:1	225	68	7.0	6.8
MgH_2_	5% V_2_O_5_	Ar	30	200	60:1	240	78	6.0	5.8

**Table 6:** Effects of carbon materials on the hydrogen storage properties of Mg-based hydride nanocomposites [85,86,87,88,89].

Hydride	Carbon Additive (wt.%)	Milling Time (h)	Milling Speed (rpm)	Ball-to-Powder Ratio	Dehydrogenation Temperature (°C)	Activation Energy (kJ/mol)	Hydrogen Capacity (wt.%)	Reversible Capacity (wt.%)
MgH_2_	5% graphite	10	400	30:1	300	108	6.5	6.2
MgH_2_	10% CNTs	2	500	20:1	275	102	6.0	5.8
MgH_2_	5% graphene	5	400	40:1	250	91	6.5	6.3
MgH_2_	2% C60	10	300	50:1	265	97	6.8	6.5
Mg_2_NiH_4_	10% CNTs	25	200	60:1	220	83	3.2	3.0

**Table 7:** Effects of metal hydrides on the hydrogen storage properties of Mg-based hydride nanocomposites [92,93,94,95,96].

Hydride	Metal Hydride Additive	Composition (mol%)	Milling Time (h)	Milling Speed (rpm)	Ball-to-Powder Ratio	Dehydrogenation Temperature (°C)	Activation Energy (kJ/mol)	Reversible Hydrogen Capacity (wt.%)
MgH_2_	LiBH_4_	5% LiBH_4_	1	400	20:1	225	98	8.0
MgH_2_	NaAlH_4_	30% NaAlH_4_	2	500	30:1	250	102	5.5
MgH_2_	TiH_2_	10% TiH_2_	5	300	40:1	275	115	6.0
MgH_2_	CaH_2_	5% CaH_2_	10	200	50:1	280	121	5.8
Mg_2_NiH_4_	LaNi_5_	10% LaNi_5_	20	250	60:1	240	95	3.0
